# Using a theoretical framework to inform implementation of the patient-centred medical home (PCMH) model in primary care: protocol for a mixed-methods systematic review

**DOI:** 10.1186/s13643-022-02132-x

**Published:** 2022-11-22

**Authors:** Deniza Mazevska, Jim Pearse, Stephanie Tierney

**Affiliations:** 1 Health Policy Analysis, PO Box 403, St Leonards, NSW 1590 Australia; 2 Radcliffe Primary Care Building, Radcliffe Observatory Quarter, University of Oxford, Woodstock Road, Oxford, OX2 6GG UK

**Keywords:** Patient-centred medical home, Implementation, Theory, ‘Best-fit’ framework synthesis, Protocol

## Abstract

**Background:**

The patient-centred medical home (PCMH) was conceived to address problems that primary care practices around the world are facing, particularly in managing the increasing numbers of patients with multiple chronic diseases. The problems include fragmentation, lack of access and poor coordination. The PCMH is a complex intervention combining high-quality primary care with evidence-based disease management. Becoming a PCMH takes time and resources, and there is a lack of empirically informed guidance for practices. Previous reviews of PCMH implementation have identified barriers and enablers but failed to analyse the complex relationships between factors involved in implementation. Using a theoretical framework can help with this, giving a better understanding of how and why interventions work or do not work. This review will aim to refine an existing theoretical framework for implementing organisational change — the Consolidated Framework for Implementation Research (CFIR) — to apply to the implementation of the PCMH in primary care.

**Methods:**

We will use the ‘best-fit’ framework approach to synthesise evidence for implementing the PCMH in primary care. We will analyse evidence from empirical studies against CFIR constructs. Where studies have identified barriers and enablers to implementing the PCMH not represented in the CFIR constructs, we will use thematic analysis to develop additional constructs to refine the CFIR. Searches will be undertaken in MEDLINE (Ovid), Embase (Ovid), Web of Science Core Collection (including Science Citation Index and Social Science Citation Index) and CINAHL. Gaps arising from the database search will be addressed through snowballing, citation tracking and review of reference lists of systematic reviews of the PCMH. We will accept qualitative, quantitative and mixed methods primary research studies published in peer-reviewed publications. A stakeholder group will provide input to the review.

**Discussion:**

The review will result in a refined theoretical framework that can be used by primary care practices to guide implementation of the PCMH. Narrative accompanying the refined framework will explain how the constructs (existing and added) work together to successfully implement the PCMH in primary care. The unpopulated CFIR constructs will be used to identify where further primary research may be needed.

**Systematic review registration:**

PROSPERO CRD42021235960

**Supplementary Information:**

The online version contains supplementary material available at 10.1186/s13643-022-02132-x.

## Background

Chronic diseases account for a large and growing proportion of disease burden worldwide [1]. In high-income countries, two out of every five people are estimated to be experiencing two or more chronic diseases concurrently [2]. This is set to rise as lifespans increase. Primary care is best placed to support people with chronic disease given its focus on generalist, whole-of-person care [3, 4]. Nevertheless, in many countries, primary care does not work as well as it should. Fragmentation, lack of access and poor coordination are key issues [5–7].

The patient-centred medical home (PCMH) model was conceived to address problems with primary care [8]. It aims to achieve the ‘quadruple aims’ of improved health outcomes for patients, improved experiences of care, reduced healthcare costs and improved provider experience of care delivery [8–10].

Examples of PCMH models across countries include the Patient Aligned Care Teams (PACT) in the Veterans’ Health Administration in the USA, the Patient Medical Home (PMH) in Canada and Health Care Homes in New Zealand. Features these models have in common include a formalised ongoing relationship between a physician and a patient, team-based care, patient-centred care, coordination, prompt access to care, preventative care and continuous quality improvement. These features tend to be enabled by payment reform (that is use of capitation or bundled payments rather than fee for service) [11, 12], regulation (including accreditation) and external facilitation and/or collaboration between practices.

Because of the multifaceted nature of the changes involved, transformation from a traditional primary care practice to a PCMH often takes years [13, 14].

Systematic reviews of the PCMH have tended to focus on outcomes of the model for patients and practice staff and not on its implementation. Where reviews have focussed on implementation, they have thematically analysed data from primary studies rather than using a theoretical framework (for example Janamian, Jackson [15], Pearse and Mazevska [16], Miller, Weir [17]). Theoretical frameworks organise explanations of how things work into descriptive categories that represent a specific theory. In implementation science, theoretical frameworks are used to understand how and why interventions work or do not work [18] and are seen as critical for successfully implementing interventions [19, 20]. Analysis of individual barriers and enablers without a theoretical framework fails to recognise the complex relationships between factors involved in implementation and their combined effect on outcomes [18].

In this review, the Consolidated Framework for Implementation Research (CFIR) will be used to synthesise evidence on implementing the PCMH. The CFIR has been selected as it is specifically designed for healthcare settings. It has also been applied to primary care change initiatives, including the PCMH. For example, Keith and Crosson [21] used it to evaluate the implementation of the Comprehensive Primary Care initiative, a PCMH model, amongst 21 primary care practices in the USA. The researchers used the tool to identify where adjustments or refinements to the intervention could be made for the participating practices for the next phase of the implementation. The current review will apply the CFIR to the large number and diverse range of PCMH implementation initiatives reported in the literature. A refined theoretical framework will be produced that can be used by primary care practices to guide implementation of the PCMH. The review will also identify gaps in studying the implementation of the PCMH that may be filled by future research.

## Methods/design

This protocol is compliant with the Preferred Reporting Items for Systematic Reviews and Meta-Analyses Protocols (PRISMA-P) guideline recommended for systematic review protocols [22]. The PRISMA-P checklist is included in Additional file 1.

### Review design

We will use the ‘best-fit’ framework approach [23–25] to synthesise evidence for implementing the PCMH in primary care practices. It is an approach to evidence synthesis that allows reviewers to test and refine an existing theory with findings from practice [25]. It involves selecting an a priori (starting) framework, so called because it need not be perfect for the research question, just ‘good enough’ [25]. The concepts represented by the framework are then used to organise data extracted from the included literature. For data that does not align with the concepts, thematic analysis is used to identify further concepts, which are then added to the framework, modifying it. A final step is to explore the relationships between the concepts in the revised framework.

There are different approaches to selecting the a priori framework for best-fit synthesis, including identifying a single framework that may be the ‘obvious choice’ for the question or topic, or a systematic search for candidate published frameworks and synthesis to achieve a single ‘meta-framework’.

As mentioned above, this review will use the CFIR [26] as the a priori framework to synthesise evidence on the implementation of the PCMH. With 39 constructs (theoretical concepts), it is the obvious choice for studying the implementation of a complex intervention like the PCMH, and it is also a meta-framework, incorporating 19 theories of implementation. Its constructs are organised into five domains (Table 1).

**Table 1:** Consolidated Framework for Implementation Research (CFIR) description of domains

Domain	Description	Examples
Characteristics of the intervention	Attributes of the intervention being implemented	Strength of evidence, complexity
Outer setting	Economic, political and social context	External policy and incentives
Inner setting	Organisational structure, politics and culture	Implementation readiness, culture
Characteristics of individuals	Attributes of the individuals adopting the intervention	Knowledge and beliefs about the intervention
Processes	Steps involved in the implementation	Planning, engaging

Source: Damschroder, Aron [26]

### Aims of the review

The review will aim to answer the question: what factors affect the implementation of the PCMH model in primary care practices and how? In answering this question, the review will also identify strategies that primary care practices have used in implementing the PCMH model to overcome barriers and to smooth transition to the model.

The outcome will be a refined theoretical model of implementing organisational change to specifically apply to implementing the PCMH in primary care.

### Stakeholder group

We will establish a stakeholder group to provide input to the review, comprised of a patient, a general practitioner, a practice manager and a practice facilitator (a role assisting primary care practices in redesigning business and clinical processes). These are roles that are either likely to use the tool arising from the review or be impacted by its use. The group will meet four times over the course of the review (Table 2)

**Table 2:** Planned meetings of the stakeholder group

No.	Timing	Purpose
1	Design phase	Receive a briefing on the review and the nature of members’ involvement
2	Before data extraction	Identify criteria to narrow the selection of included papers, if required Review the data to be extracted from the included studies
3	During the thematic analysis	Sense check the work undertaken by the reviewers
4	During data synthesis	Provide advice on the representation of the refined theoretical framework, including the diagrammatic representation and wording of the accompanying narrative

### Eligibility criteria

#### Types of studies

The review will include primary research using any study design (qualitative, quantitative or mixed methods), published in peer-reviewed publications (selected due to having undergone external review). Primary research published in grey literature (including evaluation reports), thesis/dissertation or conference abstract or presentation will be excluded. Our preliminary investigations have shown that evaluations of large-scale PCMH implementations have been published in peer-reviewed literature in addition to grey literature. Systematic reviews, editorials, commentary and opinion pieces will also be excluded.

#### Domain

The domain studied is the PCMH model of care. Papers will be accepted if they include a statement that the study is related to a practice’s/health organisation’s/health system’s implementation of a practice-level model that includes the following: a formalised ongoing relationship between a physician and a patient, team-based care, patient-centred care, coordination, prompt access to care, preventative care and continuous quality improvement. All these features need to be present for a study to be included.

#### Participants

This review will focus on stand-alone primary care practices (that is not part of a hospital) led by physicians (that is, not nurse practitioners or allied health professionals). It will also focus on primary care practices providing generalist primary care services (that is not focussing on a specific population or disease/condition). In relation to the latter, studies may report on the implementation of elements of the model for a specific population cohort (for example older people or people with diabetes), but the implementation should involve practices providing services to a range of patients and across disease/condition groups. We will exclude studies involving specialist primary care services, such as paediatric primary care, behavioural/mental health care, addiction services, cancer care, palliative care and geriatrics.

#### Intervention

Studies will be included if they report on factors affecting the implementation of the PCMH. The study need not be focussed on implementation, but to be included, it must include amongst its findings factors affecting implementation. The topic of study may be one or more features of the PCMH (such as team-based care or preventative care). However, to be included, the feature(s) must be related to a practice’s/health organisation’s/health system’s implementation of the PCMH. Studies reporting on the uptake by a practice(s) of a feature associated with the PCMH, but not in relation to the implementation of the PCMH, will be excluded.

#### Comparators

There are no comparators for this review.

#### Outcomes

The primary outcome is factors affecting implementation of the PCMH. Factors are any determinants of implementation of a PCMH feature or the model. These will be classified as barriers and enablers.

### Search strategy

Searches for this review will be through the following:

- Ovid MEDLINE (1946–present)
- Ovid Embase (1974–present)
- Web of Science Core Collection (including Science Citation Index and Social Science Citation Index) (1900–present)
- CINAHL EBSCOHost (1982–present)

The first two have been selected as general healthcare databases, the third as a multidisciplinary database and the last one is a specialist database relating to nursing and allied health, where additional studies on implementation from the perspectives of these disciplines might be found.

Search terms will relate to the setting/population (primary care) and the phenomenon of interest (PCMH). Gaps arising from the database search will be addressed through snowballing (following up references to other primary studies mentioned in included studies), citation tracking (tracking subsequently published studies that cite the included studies, using databases such as Google Scholar and Scopus) and review of reference lists of systematic reviews of the PCMH model (relevant reviews will be set aside for this purpose during the title and abstract screening). Also, any relevant study protocols identified during the title and abstract screen will be followed up to check whether the study has been published subsequently.

Where databases have a controlled vocabulary or subject heading list, this will be used, alongside keywords representing the key concepts.

Sample search terms and a search strategy using MEDLINE (Ovid) are in Additional file 2.

### Study selection, data extraction and analysis

#### Selection

EndNote X9 will be used to manage citations returned from the searches, and Covidence will be used for recording the outcomes of the title/abstract screen and full-text review.

One reviewer will screen the titles and abstracts from papers returned from the search, and a second reviewer will independently screen 20% of the papers. Where there are disagreements, the reviewers will discuss and decide jointly.

All papers that make it through to the full-text review will be reviewed independently by the two reviewers. Disagreements will again be resolved through discussion.

Depending on the number of studies selected for synthesis, to manage the review, further criteria may be applied to narrow the selection. For example, included studies may be rated on the ‘richness’ of the information that they contribute to the review question, and only conceptually rich studies were selected for synthesis.

#### Data extraction and analysis

MAXQDA, a qualitative analysis software package, will be used to code segments of the included papers and enter additional variables related to each study. Data entered/coded will include the following:

- *Information about the study*: For example, author, year, study design, country, setting and participants
- *Details about the PCMH model*: For example, features of the PCMH model, feature(s) that the study is focusing on, number of primary care practices involved, staff types involved and any special populations such as disadvantaged groups, older people or people with specific health conditions
- *Information related to quality assessment criteria*: See below.
- *CFIR constructs*: Factors affecting implementation of the PCMH relating to the constructs in the CFIR, organised into barriers and enablers, and how the constructs work together to achieve implementation and strategies that practices have used to overcome barriers within specific constructs
- *Other data*: Other factors affecting the implementation of the PCMH element or model, not captured by the CFIR constructs. These will be analysed in a subsequent stage, described below.

One reviewer will code the data from the primary studies to the appropriate fields in MaxQDA and enter additional information about each study. The same reviewer will code any data relating to implementation that cannot be mapped to the CFIR constructs into a separate ‘miscellaneous’ category. The second reviewer will progressively review the coding of the first reviewer and suggest alternative or additional ideas for consideration, which will be discussed between the reviewers as the coding develops. The second reviewer will also focus on any unpopulated CFIR constructs to ensure that they have not been missed in the coding process.

Thematic analysis will be used to develop additional constructs from the data coded to the ‘miscellaneous’ category, using the approach detailed by Braun and Clarke [27]. This will be done by one reviewer; however, another reviewer will sense check the work of the first reviewer and raise alternative views for discussion.

The unpopulated CFIR constructs will be used to identify where further primary research may be required and explore the potential for publication bias.

### Quality assessment

The mixed-methods appraisal tool (MMAT) [28, 29] will be used to assess the methodological quality of the included studies. The MMAT allows appraisal of heterogenous study designs within the single tool.

The results of the quality assessment will be used to test the synthesis (see ‘Testing the synthesis’). Lower quality studies will be ones without a ‘yes’ response to one of the two screening questions for all study types or two or less ‘yes’ responses from the five related to the study design.

### Data synthesis

The review will use a data-based convergent synthesis design as per the typology outlined by Hong et al. [30]. This approach analyses qualitative and quantitative findings together using the same synthesis method (qualitative or quantitative) and presents them as a single set of results. For this review, we are using a qualitative synthesis approach. Hence, quantitative data will be transformed into themes against the CFIR constructs or other categories developed using the thematic analysis if the data does not fit the CFIR constructs.

The CFIR will be modified using the additional categories identified during the thematic analysis of the residual data not able to be mapped to the existing CFIR constructs. Narrative of the results of the synthesis will be accompanied by a diagrammatic representation of the revised framework, showing the differences from the CFIR.

### Testing the synthesis

Carroll and Booth [24] suggest that a final step in a best-fit framework synthesis is to test it by comparing the a priori framework with the framework arising from the synthesis. This means explaining any unpopulated constructs from the CFIR as well as explaining the added constructs/refinements. Differences between the two may be due to the differences between the PCMH and implementations of other types of interventions (namely the types on which CFIR has been based) or publication bias.

Further testing of the synthesis will be through removing lower quality studies to see if their omission makes a difference to the new framework.

## Discussion

Systematic reviews of the effectiveness of the PCMH [31–36] and individual studies [37–42] show mixed results. Sinaiko and Landrum [36] comment as follows:

… Recent work identifies five domains to consider when interpreting findings of practice transformation: the practice setting, the organizational setting, the external environment, the implementation pathway, and the motivation for transformation. **Understanding which specific components of the PCMH contribute most to success is critical to determining how to invest resources in primary care transformation**.

Essentially, the authors are arguing for a theoretical framework for implementing the PCMH, identifying the critical components for successful implementation and how they work together to achieve this. The CFIR is a good starting point for identifying constructs that may be important for implementing change in primary care practices. However, the CFIR is a generic tool, and it is necessary to see how it specifically applies to implementing the PCMH model. The best fit framework synthesis will offer an opportunity to test and refine the CFIR as well as a systematic way to organise the evidence from primary studies.

Any amendments made to this protocol when conducting the review will be outlined in PROSPERO and reported in the final manuscript. Results will be disseminated through publication in a peer-reviewed journal.

## Supplementary Information

**Additional file 1.** PRISMA-P 2015 Checklist

**Additional file 2.** Ovid Medline search terms (1)

## Data Availability

Not applicable

## Abbreviations

CFIR: Consolidated Framework for Implementation Research
CINAHL: Cumulative Index to Nursing and Allied Health
PCMH: Patient-centred medical home
PRISMAP: Preferred Reporting Items for Systematic Reviews and Meta-Analyses Protocols
PROSPERO: International prospective register of systematic reviews
