# Supplementary material for: Using a theoretical framework to inform implementation of the patient-centred medical home (PCMH) model in primary care: protocol for a mixed-methods systematic review
Source: Syst Rev. 2022 Nov 22;11:249. doi: 10.1186/s13643-022-02132-x (PMC9682798; doi:10.1186/s13643-022-02132-x)
Supplement: Supplementary file 2 — Additional file 2. Ovid Medline search terms (1) [file 13643_2022_2132_MOESM2_ESM.pdf]

Using a theoretical framework to inform implementation of the patient-centred medical home (PCMH) model in primary care: protocol for a mixed methods systematic review

Additional file 2: Search strategy

Database: OVID Medline

1	primary health care/
2	community health services/
3	(primary care or primary health care or community care or primary medical care or primary health service* or general practice or family medicine or family practice or family health practice or general practise or family practise or family health practise).mp.
4	or/1-3
5	(pcmh or patient centred medical home* or patient centered medical home* or medical home* or health care home* or healthcare home* or health-care home* or health home* or primary care home* or patient aligned care team* or patient-aligned care team* or advanced primary care or enhanced primary care or augmented care or augmented service* or comprehensive primary care).mp.
6	4 and 5
7	limit 6 to (english language)
8	exp animals/ not humans.sh.
9	7 not 8
